# Anatomy, Physiology, and Pathology of the Skin

**Published:** 1825-01-01

**Authors:** 


1. *Lectures on the General Structure of the Human Body, and on the Anatomy and Functions of the Skin. Delivered before the Royal College of Surgeons, of London, in the Courses for* 1823. By THOMAS CHEVALIER, F.R.S. T.S.A. &c. Surgeon Extraordinary to the King, and Professor of Anatomy and Surgery to the College. 8vo. pp. 277, plates. 1824.

2. *Descriptions des Maladies de la Peau Observées a l'Hôpital Saint-Louis, et Exposition des Meillieurs Methodes suivies pour leur Traitement.* Par J. L. ALIBERT, Medecin de cet Hôpital, &c. &c. Avec figures coloriés. Paris, 1814–15.

3. *A practical Treatise on Diseases of the Skin, comprehending an Account of such Facts as have been recorded on these Subjects, with original Observations. The whole arranged with a View to Illustrate the Constitutional Causes of these Diseases, as well as their Local Characters.* By SAMUEL PLUMBE, Member of the Royal College of Surgeons of London, of the Medico-chirurgical Society, &c. 8vo. p. 392, two plates. London. Underwoods, June, 1824, (price 16s.)

The skin, while it is the most extended organ in the human body, is very curious and complicated in its structure—very diversified in its functions—widely related in its sympathies—and prone to numerous diseases. So numerous, indeed, are its diseases, at least in appearance, that they have been made to cover nosological charts large enough for the study of a whole life!—Even the *perusal* of Willan and Alibert is a task of no trifling difficulty; for although the eye may be amused with the *beauty of deformity* in the plates, the memory and judgment are distracted, fatigued, and ultimately dispirited by the innumerable distinctions without difference—or differences without use.—Yet the study of the skin, in regard to its structure, functions, and disorders, is extremely interesting, and not more interesting than useful. We must not, therefore, allow the difficulties of the study to deter us from the pursuit of it—for after all our embarrassments, disappointments, and fatigues, we shall reap ample reward for the time and attention bestowed on the subject.

### I. ANATOMY.

Mr. Plumbe has very properly observed, that a great defect in most writers on dermoid diseases is, "the absence of due consideration of the anatomy and physiology of the skin." He has himself, however, dedicated only a very few pages to these curious and important subjects—probably from a fear of extending a work which it was a great object to keep within restricted limits. We shall begin with the—

1. *Cuticle.* This alone has occupied several of Mr. Chevalier's lectures; and although this ingenious and lamented surgeon has amplified his subject beyond all reason, by a profusion of unnecessary, and too often affected verbiage, yet we are inclined to agree with him in most of his ideas respecting the structure and functions of this curious and important portion of the body's covering. By many anatomists the cuticle is considered as an inorganic, insensible—in fact, *unliving* structure—a mere indurated secretion from the vessels of the true skin. But to this opinion we cannot subscribe. The very circumstance of its remaining in the most intimate contact with the subjacent *living* tissue, is, to us, a proof that it possesses some degree of vitality itself—at least, in its under surface. We are inclined to the opinion of Chaussier, that, as it approaches the true skin, it becomes endued with a *shade of organization.* "Car à sa surface interne il offre encore une sorte de tissu spongieux un peu humide, qui permet de lui supposer de ce coté *une ombre d'organization* d'autant plus marquée qu'il se rapproche plus du derme." It is the most imputrescible of all the soft parts of the body, and, as Sir James Smith has well observed—"it forms a fine, but essential barrier between life and destruction, both in animals and vegetables." "Tact," says Mr. Chevalier, "in all its gradations; temperature, in its different influences and changes; absorption and exclusion, in their numberless variations, and under an infinite diversity of circumstances; perspiration, in all its degrees, take place through its well-fitted and appropriate structure"—a structure which he observes, is subjected to the domination of the principle of life—"and constituting a large and distinctly organized expansion." Mr. C. considers the cuticle as nourished by vessels ramifying on the cutis. It is constantly in a state of reproduction and decay—its exterior surface dying off in furfuraceous scales or minute particles, while its interior portion is renovated to supply the waste. We all know how profusely perspiration will pour through this structure—and how readily substances will be absorbed into the body through the same—and yet, strange to say, no pores can be seen by the best glasses. With the hope of clearing up this mystery Mr. Chevalier made some microscopical observations.

"I took some portions of cuticle from various parts of the body, and laid it in the field of a microscope, magnifying 140 times, with some glazed dark blue paper under it, hoping to detect the pores, by the coloured paper appearing through them; but I could find none that could be properly called by that name, except where it was evident it had been perforated by small hairs, which it had quitted. Instead of pores, I found an infinite number of minute velamina, regularly arranged, of exquisite tenuity, presenting a follicular appearance, and separated from each other by bands of a thicker substance, crossing and intersecting them, so as to render them distinct, as represented in the drawings, which have been made by my son." 134.

"The mystery now appeared approaching to a solution; for if the terminal vessels of the cutaneous apparatus, of which I shall have more to say in a subsequent lecture, are lodged, as I fully believe them to be, in these velamina, (of which, though perfectly distinct, I attempted in vain to count the number in the fortieth part of a square inch,) so long as the vessels maintain a vital connection with them, they transmit their secretion through them, as through a bibulous, and exquisitely hygrometrical covering, of the finest delicacy and perfection; while, through the same medium, and dependent on subjacent tubes taking a contrary course inward, absorption is carried on to a great, but less certain extent and continuity. The whole purpose which could be answered by pores, or holes, as the term is commonly understood, is thus fulfilled by an arrangement, which, while it answers all the purposes, avoids all the inconveniences of perforatory pores, as it obviates all chance of extravasation within, of hurtful exposure without, and of confusion in either direction." 135.

When this vital union is destroyed, as in a blister, for instance, the cuticle becomes incapable of continuing its transmissive office with any certainty or regularity, being reduced to its merely chemical and mechanical properties of endurance.—Hence the accumulation of fluid under its surface. Mr. C. thinks that this structure is not without analogy in the body. "The inhalation and exhalation which are constantly going on in the air-cells of the lungs, appear to take place in a manner almost or precisely similar."

The functions or uses of the cuticle have been already alluded to, and are admitted to be most important, even by those who deny it vitality or organization. Its appearances and density vary exceedingly according to the perpetually changing duties of the parts it covers, without interrupting the two grand purposes of unceasing protection and of transpiration. The hands of the black-smith or glass-melter and the delicate sempstress or watch-maker, will afford contrasting examples of cuticular variety and adaptation.

2. *Rete Mucosum.* It is now fashionable to deny the existence of the *rete mucosum*—excepting in the negro. We would ask what becomes of this part in the offspring of the negro and white? This question alone, we think, may assure us that, however difficult of demonstration in the *latter*, it still exists there, as well as in every shade of colour from Albino to Ethiopean. Mr. Plumbe offers the authority of Lawrence and Gordon (and he might have added Bichat) for the non-existence of the rete mucosum in the white. Mr. Chevalier is of a different opinion.

"It has been by some considered as a lamina of the cuticle itself; but it is no such thing; being altogether different in its ostensible properties, and intended and adapted for very different uses. It should rather be called the second, or interior Epidermis, for reasons I shall presently explain. It varies much in its colour in the natives of different countries, from the fair Circassian to the dusky Negro; and considerable variety is also evident in persons of different temperaments, inhabiting the same climate, and exposed to similar circumstances of parentage and habit. It is in general readily separable, with due care, from the cuticle. It is much more equable in its thickness, softer in its composition, and seems placed as a more certain, yielding, and delicate intermedium between the variable and insensible cuticle itself, and the vascular and nervous and sensible substance of the true cutis. In consequence of this, it serves at once duly to graduate and preserve the sensibilities of the cutis, and to secure the regular and appropriate performance of its numerous functions." 145.

Malpighi considered this body as a mucous substance secreted by the papillæ of the subjacent dermis, and serving as a kind of soft varnish in which resided the colour of the skin. This opinion has been adopted by Sabatier, by Cuvier, and by others. But Bichat denied this piece of anatomy. On separating the epidermis from the dermis he could find no rete mucosum—and therefore concluded that when this body appeared after maceration, it was the effect of decomposition. He maintained that the supposed rete mucosum was a net-work of vessels formed by innumerable ramifications and anastomoses of those which traversed the corion—and that these vessels, sanguineous and lymphatic, constituted, on the surface of the corion, (intermediate between this last and the epidermis) a capillary system, the seat of exhalation and absorption, and the secretory organ which furnished a fluid that gave colour to the skin. "Que ce système capillaire soit à la fois le siege des exhalations et des absorptions, et en meme temps l'organe sécreteur du fluide particulier qui constitue la substance qui colore la peau."—After Bichat, M. Gall examined this part, and pronounced it to be constituted of cineritious, nervous, or medullary matter destined to give origin to the convergent nervous fibres, &c. "Il veut que cet enduit grisatre, signalé par Malpighi, M. Cuvier, et autres, et nié par Bichat, soit de la substance nerveuse grise, destinée la, comme a toutes les peripheries des divers systêmes nerveux à donner naissance aux fibres nerveuses convergentes qui retournent sur la ligne mediane, vers la premiere origine des systêmes nerveux des sensations tactiles, à celle du coté opposée pour former des commissures." More recently M. Gaultier has published a memoir on the rete mucosum, in which he not only advocates its existence, but attempts to shew that it is composed of four different layers which he has minutely described. We have brought forward these opinions of eminent anatomists to shew that, however divided they may be respecting the structure of the rete mucosum, they all agree (for we cannot exclude entirely Bichat) in its existence.

But to return to Mr. Chevalier. This gentleman has criticised the opinions of Bichat, and accused him of inconsistency in his account of the rete mucosum. He cannot account for its being black in the negro and white in the European, no more than he can account for bile being yellow in an ox and green in a turtle. "It affords, he thinks, a soft nidus for the vascular and nervous structures of the complicated organ which it invests, both under pressure, and exposure to changes of temperature. It adds to the smoothness, softness, beauty, and delicacy of that organ; preserving its sensibilities, and the balance of the circulation in its extreme vessels, with more perfect precision than could be secured without it." The removal of it from any part of the surface is always followed by much inconvenience, and sometimes requires the suppurative process to secure its restoration, when the detachment of the cuticle alone could be repaired by a much simpler process, and in a manner less expensive to the constitutional powers. The various degrees of intensity with which blisters act, show all these circumstances, especially in children and persons of delicate constitution, on whom, in general, they ought not to be allowed to remain longer than is sufficient to elevate the epidermis. If the rete mucosum be also separated, especially from an extensive space, it is no uncommon thing for the vascular and even the coriaceous surface to slough. Mr. C. has seen many lives lost from this occurrence.

3. *Dermis, or True Skin.* The structure of this portion has given rise also to great disputes and diversities of opinion among anatomists. Till Malpighi's time, very ridiculous and erroneous views had been entertained of this structure; and since his time, his descriptions and opinions have very generally prevailed. That great microscopic observer divided all that was under the epidermis into three laminæ, viz. the rete mucosum—the papillary structure—and the corion, or hide. The papillary apparatus was considered by Malpighi as produced by the extremities of the nerves, which, after traversing the innumerable holes or pores of the subjacent corion, were formed into the papillæ or organs of tact. Some anatomists considered this papillary structure as entirely composed of nervous matter, and therefore, the organ of sensation only:—but others averred that into the composition of the papillæ entered the extremities of exhalents and absorbents, and consequently that it united the functions of feeling, perspiration, and absorption. But we must hear what Mr. Chevalier has to say. He begins with the vascular system of the skin.

He had discovered many years ago, that all the vascular surface of the cutis was minutely pencillated or villous—but did not consider this of any consequence till he more recently discovered the *velaminous* structure of the cuticle. In the cuticular laminæ, then, lined and assisted by the rete mucosum, "the penicilli or villi terminate, or are embedded, and carry on their ultimate duties."

"This structure is supported underneath by a perfect net-work, of more obvious construction, and of more easy detection; which, though so minute as to be seldom recognizable distinctly by the naked eye, is in reality composed of numerous trunks of vessels, for the regular supply of the ultimate arrangement. Of these, the anastomoses are innumerable: the plan is perfect. The disturbance of one part of this highly organic surface is, in a state of regular health, instantly supplied by the coadjutorship of that in its immediate vicinity; so as not greatly, or long, to interrupt the discharge of its functions. All this fine and well-guarded apparatus is spread upon the corium, and may be compared to the colours of the skilful painter, blended upon his canvass, which at once not only manifests their combination, but secures the final effect of their requisite proportions. This net-work is supplied by larger vessels underneath, which, at acute angles of various degrees, permeate the corium, after having partially ramified underneath it; then subdividing, so as thus to secure, as far as the requisite tenuity of the structure will allow, the regularity of the circulation, and the accurate fulfilment of those other functions, which are made to be dependent upon it." 161.

Mr. Chevalier is not prepared to state, from any calculation, how large a proportion of the blood may be continually flowing over the cutaneous surface. It must, however, be very considerable. Here Mr. Chevalier hazards a conjecture respecting the use of the rete mucosum, or interior epidermis, as he calls it, which is most probably original.

"What if I should consider this interior substance, so universally found under its more firm and incorruptible defender, as constituting one wide and diffused perspiratory gland, supplied by the vascular texture I have described, and being the medium of conveyance from it of that subtle fluid to the cuticle, which then exhales through its velamina, in every degree of gradation from the tranquil and invisible vapour of perfect health and ease, to the profuse and colliquative sweat of a languishing hectic, or the third stage of a fit of ague, or the clammy and panic-striking damp of the agonies of death? How else is it that our injections can penetrate no farther, when this incessant process never ends but with the end of life itself?" 165.

Of the papillæ of the skin, we have already taken some notice. Mr. Plumbe denies their existence altogether, "except in those parts of the body in which the sense of taste and touch reside," as he has never been able to discover them by the aid of the best glasses. Mr. Chevalier is of a different opinion.

"The papillæ seem to have formerly attracted the greatest share of attention; for the more obvious papillæ of the tongue, which at its tip presents one of the most perfect organs of touch, so far, and perhaps so far only, as the delicateness of tact respecting surfaces is concerned, and the larger papillæ behind, led anatomists, naturally enough, to notice the resemblance of structure in those parts of the skin which present elongated villi after ordinary injection: but the assumption of their general existence throughout the whole surface is amply supported, not only by sensations but by facts, which occasionally reveal them in a state of morbid growth, from the most minute which are observable, to what may be called, comparatively speaking, an enormous size. (See the plate.) Much that has been written and delineated, however, respecting them, is very confused, one might almost say imaginary, or perhaps has arisen from optical deception, in examinations with incorrect glasses. They appear to arise from filaments of the cutaneous nerves, which permeate the corium from within, in the same manner as the blood-vessels, and then form, most probably in close combination with them, an essential ingredient in the villous structure of the surface. Though so minute as to be only with difficulty distinguishable in a natural state, yet from their enlargement in disease, and from the analogy of their structure with that of larger animals, I believe they have generally, if not universally, a conical form; that each has an artery appropriated to its own nutrition, which accompanies it to the termination of its point in the epidermis interior." 171.

Between these recent professed writers on dermology we cannot presume to decide. Almost all the Foreign writers, from Malpighi to Gaultier, are in favour of Mr. Chevalier's opinion; and when we consider that the sense of "touch" is possessed by every part of the skin, though not in all parts so exquisitely as in the tongue and fingers' ends, we think that analogy is in favour of the existence of the papillary structure generally.

*Pores.* When the epidermis and rete mucosum are taken off, innumerable foramina, of various characters, are seen in the corion, and have been usually considered as giving outlet to the perspiration. This office Mr. Chevalier questions, of course, as he has viewed that important function under a different mode of effectuation.

"The orifices, called pores, are chiefly distinguishable in the corium, and are of three kinds. First, the residences of the bulbs of the hairs; secondly, those of the capilluli, or down, as it is commonly called; but which is, in reality, a congeries of very minute hairs, possessing similar, though not identical, characters and offices; and, lastly, those of the sebaceous glands. Here and there the places of transit of vessels and nerves make their appearance; but these, in number and amount, are very few compared to the rest, and are readily distinguished by the order, or, perhaps, I should rather say, by the uncertainty, of their arrangement.

"The orifices indicative of the situations of the sebaceous glands are not always permanent in the corium when dried; neither are those certainly so of the minuter hairs, constituting the pubescence. Those of the larger hairs, like those of quadrupeds, remain, and are easily recognizable after the process of tanning. But should we look to any, or to all of these, for an account of the ordinary, or extraordinary perspiration, they could not explain it." 181.

Mr. Chevalier next draws the attention of his hearers and readers to the sebaceous glands, as they appear "to be first, in order of function, in the growth and distinction of the maturing fœtus." Toward the period of parturition these glands are more numerous than after birth, and in our author's opinion, the great utility of these glands, during utero-gestation, consists in their secretion of a clammy and somewhat unctuous substance repulsive of water, and which secures the cuticular covering of the fœtus against the effects of long maceration in the liquor amnii. Of these sebaceous glands Mr. Chevalier avers that there are two sets—one, those well known and situated beneath the second epidermis (rete mucosum)—the other (not before detected) very minute, and lying between the rete mucosum and cuticle to which they appear firmly attached.

"I found these on examining the inner surface of some cuticle which had been macerated for more than six weeks, and from which the interior epidermis, having been broken down by putrefaction, was readily washed or rubbed off. These glands remained behind, and are clearly visible in the preparations I have made; entirely separate and distinct from the bulbs, or roots, of the capilluli." 184.

That these glands have a similar function to that of the deeper seated set, Mr. Chevalier is further convinced by a remarkable case of a young lady who consulted him some years ago, and in whom there were countless numbers of them in a diseased state about the left arm, breast, and back. They were constantly turgid with the sebaceous secretion, and were productive of great irritation. The younger Mr. Chevalier has counted 140 of these exterior glands in a quarter of a square inch, which would make them amount to nearly 120 millions on the whole surface of the body.

"The *deeper seated* set, as I have observed, reside behind the inner Epidermis, cherished, supplied, and influenced by the reticular and villous texture of the blood-vessels and nerves, and attached to the coriaceous part of the integument underneath them. They have sometimes been called miliary glands; their minuteness and rotundity giving them, as was thought, a resemblance to grains of millet-seed. It is better to denominate them from their office in the animal economy, and to preserve the term sebaceous, or, as I have called them, ceraceous, as more indicative of the nature of their secretion. Not, however, that it is either suetty, or readily inflammable; for the matter they supply is of their own distinct production and character; but in several of its uses and properties it bears an analogy to those, which we frequently derive from fatty, or waxy matter, in the ordinary businesses of life. It repels aqueous fluids, so as to call for the employment of saponaceous substances, which incorporate with both, readily to cleanse the surface which it overspreads. To a certain degree it prevents the dangers of friction, without diminishing sensibility. It is curious to observe how the tip of the nose of the sucking infant, its lips, both within and without, and the contiguous parts of the chin and cheek, are studded with these glands, to enable it to nestle, as it were, with impunity, on its mother's breast." 188.

In after life, the smoothness, softness, and polish of the skin, and its ability to bear exposure depend greatly on the regularity of secretion in these glands.

Mr Plumbe denies the existence, or at all events, the functions of the sebaceous glands. Instead of glands secreting an oleaginous or lubricating fluid, he describes a follicular apparatus.

"The cutis," says Mr. Plumbe, "on the vascular energies of which the production and nourishment of the cuticle depends, besides its more obvious and general offices as a covering to other parts, and as the structure effecting the separation of the perspirable matter from the mass of blood, gives in different parts of the body a seat to a most important structural arrangement, on the disorder of which some of the most obstinate cutaneous affections are found to depend :—the sebaceous follicles. These follicles are minute thimble-like depressions in the substance of the cutis. The larger kind are most numerously distributed over parts much exposed, and where flexures of the skin are formed; the secretion poured out in the former instance, probably forming a defence to the cuticle, under exposure to heat; and in the latter, operating to prevent the consequences of attrition. They are most easily distinguishable about the nose and mouth in men, as well as in females; but in the latter, they are also often seen in great numbers on the neck and upper parts of the chest: their secretion, which is entirely supplied by the vessels of the cutis, gives an agreeably smooth and glossy appearance to the skin of these parts, where their dimensions and number are not very considerable; but where the reverse is the case, the secretion at their orifices becomes discoloured, forming so many minute black spots, which much disfigures these parts, and gives them a dingy, unhealthy appearance." 4.

Mr. Plumbe thinks that Mr. Chevalier has been deceived respecting these glands—and that the little white pearly prominences described by him as glands, and illustrated by a preparation deposited in the College Museum, are no other than "concretions of sebaceous matter on the mouths of the follicles, retained in that situation by the cuticle." On maceration, he observes, the cuticle peels off, leaving the concretions undisturbed. In the preparation alluded to, many of these specks are already detached and floating loose in the spirit—an occurrence which, he thinks, would not have so soon taken place, had they been such as Mr. Chevalier describes them. Mr. Plumbe's apparatus and opinions have been anticipated by Chaussier and others. This gentleman distinctly describes the sebaceous follicles. "Enfin, le derme offre dans toute son etendue et dans toutes les areoles de son tissu propre un grand nombre de follicules destinées à secreter une humeur huileuse qui entretient la souplesse de la peau."*

*Capilli and Capilluli.* Mr Chevalier differs from the best dermologists in respect to the seat of the *bulbs* of the hair.—He places them on the exterior surface of the corium, in which they are embedded, and from whence the hairs spring out obliquely through the rete mucosum and epidermis, the latter embracing them very firmly as they emerge from the surface.—Mr. Plumbe, and the continental dermologists are all agreed that the seat of the bulbs is below the inner surface of the cutis—in fact, in the cellular membrane. "Il (le bulbe) est situé presques toujours dans le tissu cellulaire sous-cutané et quelquefois *plus superficiellement* dans les areoles qui se voient à la *face profonde* du chorion dans l'epaisseur duquel, au premier coup d'oeil, il parait alors implanté." *Villermé.*

In man the hairs are tubular, the tubes being intersected by partitions, resembling the sap-vessels in plants. Whether the hairs transmit any secretion, Mr. C. thinks is worthy of enquiry.

*Corium or Dermis.* This is the firm stratum of the skin which forms the basis, on which the other parts are, as it were, spread out. Its structure appears chiefly made up of a fibrous substance, the threads of which are interwoven with each other in various directions, so as to give a compactness that remains long after death. It is pierced by innumerable holes for the transmission of the hairs, the nerves, and the blood-vessels—but has very few blood-vessels of its own—only those to nourish its proper substance. Immediately underneath it, the cutaneous arteries are distributed in an arborescent form, anastomosing freely, so as to secure the regular supply of the reticular and villous texture—their ultimate destination. "Here they are retained by the cellular membrane, into which the structure of the corium itself seems gradually to subside;—so that it is sometimes a nice point to distinguish where one absolutely ends, and the other begins." *Chev.*

* See vol. 8 of Dict. des Sciences Méd. Art. *derme.*

We trust that this slight anatomical sketch of the different structures composing the skin, will not be unacceptable to our readers, as a reference when the subject is under discussion. It has been drawn from sources which are not at the command of even the generality of our readers, and we have taken some pains to compress the known and ascertained facts into as small a space as possible.

Mr. Chevalier's work will not come again under notice in this article, and that respectable surgeon is, himself gone to the "house appointed for all living," since its publication. This last circumstance must be our apology for not indulging in any criticisms on the work of one who is no longer able to answer for himself. We do not wish to be classed among those hyenas who tear open the graves of their victims, to glut on the mangled and quivering members! Quiescunt in pace. But even were Mr. Chevalier alive, we should have but few strictures to make on his work. It's greatest and almost only fault is excessive dilution of the matter by a most extraordinary and unnecessary redundancy of words, metaphors, and allusions. We will give a single example, which is indeed a fac simile of the whole.—Thus, for the simple expression—suppression of perspiration, we have the tiresome periphrasis—"suppression *of* the actions *of* the organ *of* perspiration." P. 199. Still, as we said before, the matter itself is unobjectionable when freed from the prodigious load of words in which it is buried.

We have said little or nothing on the functions of perspiration and absorption. It is evident that no separate or distinct apparatus can be discovered for either process—and therefore, it follows that both functions are performed by the same vessels (as far as we know)—that is, by the vascular system of vessels spread over the outer surface of the corion. The *modus* is a mere matter of conjecture. The fluid thrown off by the cutaneous vessels is of such various appearance and qualities, that no rational thinker can view it in any other light than as a *secretion;* and therefore, we think Mr. Chevalier perfectly justified in characterising the skin as an immense gland, destined for a most important secretion—and uniting in itself the other functions of feeling and absorption. In this triple capacity it exercises a most powerful influence on the other and interior functions of the body—forming the centre of a train of sympathies the most complicated and wonderful—and becoming in its turn the *subject* of reaction from within, as will be abundantly seen in the host of cutaneous diseases to which we are next to turn our attention.

We now come to the pathology of the skin, and more particularly to Mr. Plumbe's work. This gentleman has for some time paid attention to cutaneous diseases, and is well-known as the author of a work on porrigo, already noticed in this journal. In the *arrangement* of his subjects, Mr. Plumbe has endeavoured to obviate inconveniences that have been often complained of in classifications founded merely on local character, although he is conscious that it would be nearly impossible to construct a classification, that would completely comprehend both the local characters and constitutional causes under precise definitions. Still he thinks that, in a large proportion of the divisions he has instituted, a step of some utility will be gained. The study of cutaneous diseases he considers as yet in its infancy; and he will not admit that the enquiries and researches of Dr. Willan among the writings of ancient authors have elicited any useful information—no great encouragement to future bookworms! These are nearly the sentiments of Alibert. "J'entre," (says that illustrious but somewhat vain dermologist) "dans une carrière presque deserte, où peu d'hommes ont penetré avant moi; où aucun travail anterieur ne m'a servi de guide; où tout est nouveau pour l'observation; où tout est problême pour la pensée." A little farther on M. Alibert affirms that "the works which we possess (on cutaneous diseases) offer nothing but a mass of confused ideas, of dangerous or unnecessary theories." The picture which M. Alibert draws of the endless varieties of cutaneous defædations is enough to frighten most men from the attempt to study them. "Quelle inconcevable variété dans les degradations de tont geure dont nos tegumens sont susceptible!" Mr. Plumbe has endeavoured "to steer as widely as possible of those numerous divisions into species where slight variations only exist, and those endless distinctions without real differences, which have been made by this author (Dr. Willan) as being calculated to discourage the student rather than promote the knowledge of the subject."

"Abbreviation of remark, so far as it could be observed without the sacrifice of any important consideration, and an arrangement of the engravings calculated to convey clear ideas of the different diseases in one plate, have been had recourse to, with a view to escape a serious obstacle to the propagation of the knowledge of these subjects, among persons whose pecuniary means of obtaining information are at all limited." vii.

M. Alibert, in the third section of his "Discours Preliminaire," takes a philosophical view of those causes which contribute to the development of cutaneous diseases:—And as this magnificent and expensive work, is in the hands of very few on this side of the channel, we deem it useful to set before our readers the prominent features of the said section, more especially as etiology, *in a general way*, is scarcely touched upon by Willan, Bateman, or Plumbe. Alibert arranges his observations on this subject under eleven heads, and these we shall follow seriatim.

M. Alibert prefaces this section with some interesting remarks on the *modifications* which age, sex, temperament, seasons, and climates produce on cutaneous diseases. The eruptions attending dentition in infancy, and the pruritus of senescence are examples of the first of these modifying causes.—Sex has considerable influence. M. Alibert lately saw a lady who suddenly and prematurely weaned her child. Her upper and lower extremities became quickly covered with a crustaceous eruption. She applied the child again to the breast, and the eruption quickly disappeared. The influence of seasons and atmospheric vicissitudes on the skin are notorious—and that of climate, in an extended sense of the word, will be forcibly called to mind by the plica of Poland—the yaws of America or Africa—the elephantiasis of India—the lepra of Egypt—the pellagra of Lombardy—the dartre of France, and (without meaning any national reflexion) the itch of Scotland. But we now come to the more direct causes of cutaneous diseases.

1. M. Alibert has properly placed at the head of this long list, those derangements of the digestive organs produced by unwholesome or improper food and drink. The Hospital of St. Louis, is perhaps, half-peopled from this cause—or rather chain of causes.

2. Exposure to atmospheric influence is the next agent laid down by our author, as among the most active in the production of cutaneous maladies. Cold, moisture, in short, vicissitudes, check the transpiration, and this secretion accumulating in the conduits, excites a host of herpetic and erythematous eruptions.

3. *Want of Exercise.*—M. Alibert has found this a prolific source of cutaneous defœdations, among the now almost innumerable ramifications of the sedentary classes of society—high and low. Men of letters, savans, philosophers, mechanics—all suffer from this cause. The vascular system of the skin becomes enfeebled from want of air and muscular exertion—and eruptions of various kinds follow as the natural consequence.

4. *Extremes Approximate.*—Prolonged watching, fatigue, and want of rest, give rise to ulcers, and dartrous affections of a very indomitable kind.

5. *Want of Cleanliness.*—On this point we need not comment. Its influence is extensive and predominant.

6. M. Alibert has seen many cutaneous diseases clearly resulting from particular trades and professions—which have escaped the observation of Ramazini.

7. *Contagion.*—This is an abundant source of the diseases in question, but, as in other circumstances, it is veiled in great obscurity. Every cutaneous malady has, as it were, its own peculiar channel or mode of communication. He has made numerous experiments at the St. Louis on the subject of contagion. Thus, while variola and vaccina may be conveyed by the introduction of matter into the absorbents, the same cannot be done with the itch, which he has, in vain, tried to produce by inoculation. The facility of transmission, in cutaneous maladies, is greatly influenced by the physiologic condition of the skin to be acted on. Thus in a sound and vigorous state, the cutaneous surface will catch a disorder from another far more readily than if in an opposite condition. Extensive experience has also taught him that the more superficial eruptive diseases —those which are almost entirely confined, both as to cause and effect, to the skin itself, are far more contagious or transferrible, than those connected with, or dependent on deranged condition of the internal organs. It is fortunate also that the most inveterate cutaneous diseases are least contagious. Even tinea, he avers, is seldom communicated by simple contact, unless there be various circumstances to favour its transmission.

8. *Insects* take their share in the production of the class of affections under review. The itch is attributed by M. Alibert to the acarus. The dracunculus and other depredators are familiar examples.

9. *Moral Affections* of a depressing nature are powerfully conducive to cutaneous diseases. One cause why animals are less subject to these disorders than man, may be sought in their comparative freedom from mental emotions and conflicting passions. Our author has given a drawing of a servant, who, on seeing his master dragged to the scaffold, became almost instantly covered with a furfuraceous eruption; and he has seen an instance of a woman become quickly overspread with a papular eruption merely from the receipt of a piece of bad news.

10. *Manners and Customs.*—The erudite researches of Willan have shewn us how great an immunity the ancients enjoyed from cutaneous defædations. Homer scarcely mentions such a thing, except in the person of some dirty beggar. Herodotus, Thucydides, Diodorus Siculus, are equally silent, excepting some straggling traditionary instances to which they allude, as punishments on the individuals. The early Romans prided themselves on their freedom from deformities of the skin. But luxury increased—the martial and gymnastic exercises were neglected—indulgence in idleness, debauchery, and drunkenness began to prevail—and *then* cutaneous complaints became common. Neither the Germans, Gauls, nor Britons, were affected with these diseases till after their commerce with the Saracens. It is melancholy to reflect that two of our greatest scourges, Variola and Syphilis, owe their introduction into Europe, most probably, to political or commercial speculations.

11. Last, though perhaps not least, is hereditary disposition. Numerous facts indisputably prove that families become degraded or at least degenerated, by the mournful inheritance of diseases or their germs, transmitted from generation to generation. Our author has seen numerous instances of the strange modifications of cutaneous diseases produced by descent. He saw a woman, who, in pregnancy, suffered severely from confluent small-pox. Her infant came into the world covered with a scaly eruption of most hideous appearance. In another case, when the male parent was excessively afflicted with gout, the children were born with severe herpetic eruptions.* It not unfrequently happens that cutaneous diseases are transmitted from parent to progeny with symptoms precisely similar. M. Alibert saw the child of a parent, with cancer of the lip, affected with a similar disease. The two men lately exhibited all over Europe, with scaly skins, were descended from parents whose skins were also scaly. He concludes with the belief that there is no class of disease more transmissible, or, in other words more hereditary, than those now under consideration.

We have dwelt the longer on this etiological sketch, the result of unequalled experience, because it will shew that, with a few exceptions, the causes of cutaneous diseases are the same as of most other diseases, and consequently that the great majority of them are dependent on, or connected with some derangement of health—especially of the digestive organs—and consequently to be treated under that impression.

* Dr. Parry, in his inestimable work on pathology, has given numerous examples of this kind. By the bye, is the younger Dr. Parry ever to bring out the long-expected second volume of his father's work?

Mr. Plumbe has thrown his arrangement into five sections, as follow:—

Sec. I. "*Diseases which obtain their distinguishing characteristics from local peculiarities of the Skin;*"—containing ACNE and its varieties—SYCOSIS—PORRIGO, SCUTULATA—FURFURANS—and LUPINOSA.

Sec. II. "*Diseases dependent on debilitated and deranged States of System, and consequent diminished Tone of the Vessels of the Cutis*"—embracing PURPURA and its varieties—PEMPHIGUS and POMPHOLYX—ECTHYMA and RUPIA.

Sec. III. "*On Diseases exerting a probably salutary influence on the System, originally produced by, and usually symptomatic of, deranged digestive Organs, and characterized by active Inflammation;*"—comprehending PORRIGO FAVOSA and LARVALIS—STROPHULUS LICHEN—LICHEN TROPICUS—PRURIGO—URTICARIA—HERPES—THRUSH—BOILS.

Sec. IV. "*On Diseases marked by chronic inflammatory Action of the Vessels producing the Cuticle;*"—as LEPRA and its varieties—PSORIASIS—PITYRIASIS—PELLAGRA—ICTHYOSIS—WARTS.

Sec. V. This is a convenient class in all systems of nosology. It is a kind of pariar or outcast class, admitting all those diseases which can find admittance no where else; viz.—"*Diseases of a mixed character, &c.*"—including IMPETIGO—SCABIES—ECZEMA—ERYSIPELAS—ELEPHANTIASIS—ERYTHEMA and ROSEOLA—VENEREAL ERUPTIONS.

Such is the classification or arrangement of the latest writer on dermology—and we have little doubt that his cotemporary dermological practitioners and critics will be able to raise as many objections to it, as he has done to the arrangements of others. Considering it nearly as difficult, however, to arrange and portray the ever-varying eruptions on the skin as the ever-varying clouds in the sky (both of which tasks have been undertaken) we do not set a very high value on purely dermological criticisms—and therefore, shall take little notice of them, in this analytical sketch.*

* We have received some severe strictures on Mr. Plumbe's work from more than one or two anonymous sources. But of these we shall not avail

As it would be obviously improper to go through a regular analysis of an elementary compilation, like that of Mr. Plumbe's, we shall only take a single section, and that the first, containing among other diseases, tinea capitis, as an example of our author's manner and matter, which will enable our readers to form some judgment of the whole work.

### SECTION THE FIRST.

1. *Acne.* It will be seen that Mr. Plumbe commences with *tubercular* eruptions, the subject of which is taken up at page 72 (3d edition) of Bateman, and by a curious coincidence, at page 72 of Alibert. Four varieties of Acne are described by Bateman, (simplex, punctata, indurata, and rosacea) the necessity for which division is questioned by Mr. Plumbe, who therefore commences the work of reformation, or at least of simplification, at the very threshold. The descriptions are nearly the same in both authors, and need not be here repeated. Mr. Plumbe considers the eruption as arising originally from obstruction of the sebaceous matter in its passage to the surface of the skin, by which a considerable degree of hardening of that substance is produced, as well as accumulation. The simple appearance of such spots on the face should not, he thinks, be considered as a disease, or as deviation from health—" the only desirable change which can be effected, where they exist to an unpleasant extent, is that which frequent ablution and moderate friction can produce." The plate which Alibert gives of this eruption, under the title " *Dartre pustuleuse Miliaire*" on the face of a young lady of 18, would be considered by most young ladies, as a very formidable disease indeed. Alibert's

---

ourselves, as most of them bear the impress of the detestable *Odium Medicum*, the disgrace of our profession. Mr. Plumbe has been censured for his expressed contempt of what the ancient writers have said and done respecting cutaneous diseases. He is not singular in this. The master Dermologist of the age, M. Alibert, has expressed himself in still stronger terms. Speaking of Tinea, M. Alibert observes :—

" Que trouve-t-on dans les auteurs, touchant la nature et le caractere specifique des Teignes ?—Des renseignemens incertains, des dissertations vains, des détails vagues, toujours insuffisans. Il semble que les Anciens aient ecrit au hasard sur cet exantheme aussi repoussant qu' opiniâtre.—J'elague de cet ouvrage les discussions futiles aux quelles se sont livrés mes predecesseurs. Je transcris ce que J'ai observé, m'inquietant peu de ce qu'on a dit avant moi. *Quand on voit de si près la Nature, quel besoin a-t-on de recourir aux travaux des Grecs et des Arabes ?—Tout etalage d'erudition ne seroit qu'un vain jeu de l'esprit, sans avantage pour la science.*"

This is a home thrust to those who trust more to what they read in the Ancients, than what they see with their own eyes. What is the use of eyes to these last ?

patient, however, was vigorous and healthy, though much annoyed by the itching of the eruption. But under circumstances of actual and long-continued follicular obstruction, the complaint becomes troublesome, and the practitioner is called in. " The immediate exciting cause of this inflammation is usually found to depend on some accidental disorder of the primæ viæ;" but, " independent of the disposition to this affection which the habit of living may generate, some individuals are peculiarly disposed to it from *original formation of skin*, and hence has arisen the idea of its *hereditary* character." We think, this is rather eluding than meeting the question. We would ask Mr. Plumbe, whence the *original formation of skin?* Certainly from an hereditary source. In the same way pulmonary consumption has been attempted to be shewn as having no hereditary tendency. It is true, that tubercles in the lungs, or disposition to them, which amounts to pretty nearly the same thing, are transmitted from parent to child, and these tubercles sooner or later, in general, end in phthisis. But because children are *never* born with hectic fever and purulent expectoration, it is gravely and logically inferred, that phthisis is *never* hereditary. We see features, figure, and other peculiarities often transmitted by descent—and why not certain peculiarities of internal organization? We know of no just cause why they should not—indeed, we are convinced that they are transmitted, occasionally.

" When disorder of the digestive organs has been the immediate exciting cause of acne, the symptoms of the first affection are alleviated, and there is no doubt that more formidable mischief is sometimes prevented by its occurrence: in the treatment of the mild and transient kind described, therefore, the state of these organs and the general condition of the system ought to occupy the chief attention: not so much, however, from the fear of doing mischief by sedative applications in repelling the eruption, but as leading to the most direct remedial measure. Repellents, as they have been termed, seldom have any effect in preventing suppuration, and this ought to be considered the most desirable course it should take. Frequent bathing the parts with warm water, and gentle friction with the mildest kind of soap, constitutes by far the best kind of local application, its effect being at once to allay irritation, and promote suppuration, and at the same time to remove any accumulation in the follicles of the part as yet uninflamed." 23.

Mr. Plumbe avers, that, except in the old-established and indurated state of this affection, the use of stimulants, as practised by the ancients and approved of by Bateman, is obviously improper, because in opposition to the first principles of surgery. Stimulants, he says, only add to the irritation and extent of the disease, and consequently to its duration, "while the plan which conducts the tubercles to a safe and healthy suppuration is least painful to the patient, and free from danger of the supposed prejudicial effects of sedatives."

Mr. Plumbe has no doubt that blisters, as recommended by Bateman, might put an immediate stop to the disease—the vesication clearing the obstructed follicles—while the inflammation is checked by the counter-irritation in the neighbourhood. "But it is a remedy unnecessarily severe, and exceedingly inconvenient."

"The foregoing remarks apply more particularly to the states and consequences of obstruction of the sebaceous follicles, denominated acne simplex and acne punctata. When the disease occurs more extensively, and habitual disorder of the digestive organs, or scrofulous diathesis exist, a slow and unhealthy suppuration takes place, spreading from the originally inflamed follicle, and involving to a considerable extent those in the neighbourhood, producing, instead of a remote pustule, a considerable, though slowly formed, collection of matter. The latter, instead of finding its way quickly to the surface, accumulates and disorders the substance of the cutis to a great extent. Its course and extent is marked during the more active state of the inflammation by a florid looking and very irregularly formed, rather prominent, tubercle, exceedingly tender to the touch, and in some one or two points soft: still the superincumbent skin is not ruptured, and in the course of a short time it assumes a dark-blue colour. In this state it sometimes comes under the notice of the surgeon, when the existence of matter being ascertained, a lancet is thrust into it, and the contents discharged, leaving for some time an unhealthy livid edge to the orifice, which slowly heals, and, in most instances, leaves a mark of some duration on the part. If neglected beyond a certain extent, however, and this course is not taken, the matter is either absorbed in the most sluggish and protracted manner, or a small portion remains to find its way through the skin, leaving a more minute sore, which soon dries up; a blue discoloration of the spot sometimes remaining for months." 27.

In the treatment of this affection Mr. P. laments the deficiency of attention to common surgical principles. The general application of stimulants must be improper, while any vestige of inflammation exists. On the contrary, poultices and fomentations are necessary to soothe irritation, and promote suppuration. "If any tubercle should be found assuming the blue colour without signs of matter coming forward, it should be freely punctured," the matter evacuated, and irritation allayed, then stimulants may be proper. The oxymurias hydrargyri dissolved in proof spirit, in the proportion of five grains to eight ounces, is preferred by Mr. P. to other lotions. Mercurial ointment, he thinks, would be still better, but it is an unpleasant application to such a part. Alteratives and tonics internally are recommended, as there is generally some constitutional derangement.

"The chronic inflammatory redness and enlargement of the nose, which constitutes the state of acne rosacea, the affection under consideration, is always the result of repeated attacks, or of long continuation of the obstructed state of the follicles, the direct exciting cause being an overloaded state of the stomach, and frequent excessive and general excitement of the digestive organs. There seems to be a satisfactory explanation of the disease being first found to attack the nose, in the fact, that the follicles are here very thickly distributed, while it is more exposed to the chilling effects of cold, and consequently more frequently to the effects of reaction: constant checks being thereby afforded to the healthy progress of suppuration, as well as means of increasing and extending the inflammation." 33.

We shall pass over sycosis, as our author considers it neither more nor less than acne, or follicular obstruction occurring in parts covered with hair, and consequently assuming a more obstinate and formidable character. Where the hair can be extracted without pain, this, he thinks, should be a preliminary step.

2. *Porrigo.* Our author directs his principal attention to a consideration of p. scutulata, or common ring-worm of the scalp, which he affirms to be only the commencement of that disease, which, under circumstances of great neglect, answers to the description of porrigo furfurans and p. lupinosa. Having written professedly on the subject of ring-worm of the scalp, our author is much more copious, in proportion, on this than on most other individual topics. And as it is an important disease we shall make it the conclusion of this first dermological article.

Mr. Plumbe's description differs but in one item from that of Willan. His observations altogether are very much more full than those of Bateman. As Willan is in the hands of comparatively few, we shall give the following descriptive sketch from Mr. Plumbe.

"The symptom by which this disease is usually first discovered, is the falling off of the hair of the part. The attention being attracted by this occurrence, the scalp appears, on examination, to have assumed a somewhat scurfy and slightly reddened appearance. The hair remaining on the diseased part, is thin and irregularly scattered over it, the greater portion appearing to have been removed by the roots, while some have been broken off near to the scalp, the roots of which still retain their situation. Those which remain apparently growing on the part, will be found to drop off on friction, or to have, on being pulled, scarce any hold on the scalp.

"In the majority of cases, at their first commencement, these will be the only appearances noticed. The existence of the minute straw-coloured pustules, denominated achores, does not appear necessary to constitute the disease, as they are not seen for a short time later, and not till after some degree of itching and irritation of the part has been felt.

"The itching and irritation commencing simultaneously with the formation of pustules, the child who is the subject of the disease soon ruptures a few of them, and spreading, by the frequently repeated application of the nails to the spot, their contents over the adjacent parts of the scalp, extends the mischief with great rapidity upon it; the same destruction of the hair, and subsequent pustulation, marking its progress.

"When pustules are noticed, they are uniformly found with hairs growing through them; and if the disease has existed for a considerable length of time, and destroyed the greater part of the hair of the part, such pustules are found proportionately reduced in number; but still surrounding the few straggling hairs which remain; each single minute pustule appearing to be dependant on the hair in its centre.

"If the hair, as sometimes happens, be completely eradicated from the spot where the disease first appears, the skin assumes an apparently healthy character; the disease, as regards this particular spot, may be said to have exhausted itself.

"In traversing the other parts of the scalp, however, the power of infection being kept up by the secretion of the pustules of such parts, till the new hair again appears in the original situation of the disease, a recurrence of the pustules takes place here, and the same destruction of the new hair is observed, as of the old." 44.

The disease, when once formed, our author avers, spreads only to a small extent by the application of the infectious matter, *and not from the mere communication of the specific action of the vessels of the part to those adjoining*. This being the case, the importance of cleanliness becomes obvious enough—and indeed Mr. Plumbe appears to attach far more virtue to this alone, than to any medicinal application whatever, cleanliness being neglected. Mr. P. does not believe in the statements handed down to us respecting the effect of ring-worm in altering the colour and strength of the hair—unless ulcerations have been allowed to form and affect the parts whence the hair is secreted. These effects seldom are seen in decent society. When the secretion accumulates and scabs are formed, it is then denominated *scald-head*. Willan observes that the complete extirpation of the hair, is followed by a return of the natural colour and appearance of the scalp. "A remark," says Mr. Plumbe, "which appears to have been made without exciting the suspicion that the hair, while it remained, had any share in keeping up the disease." The circumstance of there being a hair uniformly growing in the centre of the pustules appears to have led Dr. Underwood long ago to suppose that tinea commences in the roots of the hair. Hence probably his practice of the pitch cap for eradicating the hair. This practice, Mr. Plumbe thinks, was founded on a just principle, though too cruel in its effects. Mr. Plumbe is rather tiresome when he gets on this subject of the hair. He seems so enamoured of it that it is with difficulty he leaves it. This is the case however with every writer who has got a hobby. The following extract contains the pith of our author's favourite pathology.

"The disease may be defined to be inflammation of a specific character, affecting the solid structure of the cutis. It will be recollected, that pustules only occur where hair exists, and that each pustule has usually one in its centre: and bearing in mind what has been already stated, as to the probable influence of the hair on the scalp, where the cutis is actively inflamed, we may be warranted in the conclusion, that the specific irritation of the disease obtains an accession of strength from the local influence of the hair, where it penetrates the scalp; and that the formation of a pustule is the consequence of such accession." 65.

The porrigo scutulata appears to be the *tinea granulata* of Alibert, who has given a fine plate of this species.

*Treatment.* The first step which Mr. Plumbe takes is to remove any hair remaining on the part, and which comes away without pain to the patient.

"This precaution is necessary, whether pustules may have formed in great numbers or not, and not only should such gentle force be used as is consistent with ease to the patient, in trying the hair of the diseased part, but that which is apparently healthy, surrounding the margin, should be submitted to the same test; for it not unfrequently happens, that the hair will easily separate a short distance from this, without pain, and lead to the discovery of the mischief to a greater extent than was first suspected. It must be taken for granted, that those hairs which will separate with a slight degree of force have lost the means of support and nourishment, either as the consequence of the formation of a pustule around them, or from the influence of the irritation of the disease, on its secreting structure which has been explained; and that their remaining sticking in the scalp can only be productive of further irritation on the part." 71.

This is done, not by the indiscriminate eradication of the pitch-cap, but by a pair of small forceps. Shaving the part, though necessary in all cases after the loose hair has been removed, is no effectual substitute for the eradication, in Mr. Plumbe's opinion. The next stept, and preliminary to any medicinal application, is to discharge the contents of as many of the pustules as possible, by pinching up the skin between the finger and thumb, and carefully washing away what is thus forced out, by warm soap and water. This done, some astringent application, possessing the power of taking from the secretion its contagious quality, and at the same time of constringing the vessels from which it flows, and lessening its quantity, may be made use of.

"A solution of the sulphate of copper has been employed in some cases for this purpose: I believe the object to be more completely accomplished, however, by rubbing this preparation, in a finely powdered state, on the part, and then washing it off." 73.

Some sedative application is necessary to keep down any temporary inflammation occasioned by drawing out the hairs and shaving the parts.

"A careful examination should be instituted every morning, and if any pustules appear, they should be at once removed, and the sulphate of copper applied, as at first, to the part. After two or three repetitions of this application, no fresh appearance of pustule takes place, and the circle of the disease is marked by small thin scabs of a darkish colour, and the same characteristics in other respects, as the common exudation from abraded surfaces to the cutis. These scabs separate in a few days, bringing with them a few of the remaining hairs which have separated, and leaving a shining red and irregular surface, which gradually loses its inflammatory character, having now and then a little scurf forming on it, till the new hair begins to appear." 74.

When a great number of pustules has been formed, the effects on the scalp are such that we need scarcely look for strong and healthy hair in a shorter period than six weeks or two months. At the end of three months it usually attains its original strength.

It is to be observed, that the treatment above described, as applicable to simple ring-worm, requires some modification in those long-established cases, where great accumulation of scabs, portions of ulcerated surface, and an irritable state of the vessels exist. Fomentations, poultices, and "pressure and cold applications combined," have rarely failed, in our author's hands to finally subdue the disease. The foregoing description of the local treatment of porrigo scutulata comprehends every thing on which our author is able to speak from experience. He condemns the use of greasy applications, as he has frequently seen the disease spread rather than subside under them. In cases of simple ring-worm, as it is usually seen in the middle and higher classes of society, where cleanliness is observed, he thinks internal remedies can rarely be necessary. But in severe cases, such as are alluded to above, the occurrence of constitutional irritation will demand constitutional remedies. Mr. Plumbe's remarks on the porrigo furfurans and lupinosa require no notice here.

"On the subject of the infectious properties of Porrigo, it is necessary to state that the advanced and neglected conditions of the disease are not possessed of this property to any considerable extent; and indeed I am not satisfied that it does not, in the course of time, lose the power of infection altogether: its specific character seeming to subside into the mere effects of local irritation upon diseased parts." 93.

Here we must close our article. Not making criticism our trade, we shall say very little on the merits or demerits of Mr. Plumbe's work. It certainly is not without some originality, and we think he has corrected some erroneous notions of more celebrated authors than himself. But, from the looseness of the style and the size of the type, he has spread the matter of his work over at least double the space which it might have occupied, had Mr. Plumbe been practised in literary composition. In the next edition (and we venture to say it will go through several) we advise him to remodel the language and style, in toto, studying terseness instead of diffusion, by which means he may nearly double the matter without adding to the size or price.

